# A unified ontological and explainable framework for decoding AI risks from news data

**DOI:** 10.1038/s41598-025-10675-x

**Published:** 2025-07-15

**Authors:** Chuan Chen, Peng Luo, Huilin Zhao, Mengyi Wei, Puzhen Zhang, Zihan Liu, Liqiu Meng

**Affiliations:** 1 Chair of Cartography and Visual Analytics, Technical University of Munich, Munich, Germany; 2 Senseable City Lab, Massachusetts Institute of Technology, Cambridge, MA USA; 3 Department of Land Surveying and Geo-Informatics, Faculty of Construction and Environment, The Hong Kong Polytechnic University, Kowloon, Hongkong China

**Keywords:** AI ethics, AI risk, Ontological model, News data, Explainable machine learning, Applied physics, Human behaviour

## Abstract

Artificial intelligence (AI) is rapidly permeating various aspects of human life, raising growing concerns about its associated risks. However, existing research on AI risks often remains fragmented—either limited to specific domains or focused solely on ethical guideline development—lacking a comprehensive framework that bridges macro-level typologies and micro-level instances. To address this gap, we propose an ontological risk model that unifies AI risk representation across multiple scales. Based on this model, we construct an enriched AI risk event database by systematically extracting and structuring raw news data. We then apply a suite of visual analytics methods to extract and summarize key characteristics of AI risk events. Finally, by integrating explainable machine learning techniques, we identify potential driving factors underlying different risk attributes. This study provides a novel, quantitative framework for understanding AI risks, offering both structural insights through ontological modeling and mechanistic interpretations by explainable machine learning.

## Introduction

AI has become integral to many domains, offering increasingly intelligent and efficient services. However, its rapid development has also raised concerns and unintended consequences, giving rise to the field of AI ethics. As AI systems take on human-like functions and roles, they inevitably confront ethical challenges similar to those faced by humans. Since AI products and algorithms cannot be categorically defined as human entities, the study of AI ethics requires a departure from traditional ethical studies centered on human subjects, and a nuanced understanding of the characteristics of AI that differ from those of humans. Given the extensive interactions between AI and humans, the study of AI ethics is more concerned with AI-human interactions rather than AI in isolation^1,2^. One of its priorities is to examine the risks associated with AI and its potential to cause harm to humans. This study aims to address two key objectives: to develop a structured and multi-scale representation of AI risks through ontological modeling, and to extract and analyze AI risk patterns from news data using visual analysis and explainable machine learning.

Two basic ethical theories are commonly used in AI ethics research: utilitarian ethics and virtue ethics^3–5^. Utilitarian ethics aims to maximize the overall good as the primary goal. In contrast, virtue ethics revolves around assessing whether each element of a set of behaviors is consistent with ethical values, emphasizing the need for transparency and interpretability in behavior. These foundational ethical theories all share a common standpoint - human interests as a fundamental value, and are committed to reducing the risk of AI directly or indirectly harming human interests.

Research on AI risks is a foundational, empirical study focused on identifying, assessing, and mitigating specific technical, security, and societal threats posed by AI systems in real-world scenarios. This groundwork provides concrete data and insights essential for the practical application of AI ethics, ensuring informed and responsible decision-making. However, research on the risks of AI is just getting started, which is also limited in the field of AI ethics. AI ethics research currently manifests itself in two primary strands^6^. The first strand focuses on formulating overarching guidelines. The second delves into specific facets such as privacy invasion and bias, offering a localized perspective on AI ethics. Ethical guidance serves as an important framework for society, helping policymakers and practitioners understand and address the ethical challenges of AI. This macro view is beneficial for governments, businesses, research organizations, and society at large. However, it lacks a granular examination of AI risks. In contrast, studies that focus on particular aspects, such as privacy invasion and bias, provide detailed insights into potential risks in specific AI domains at the micro-level. While these focused investigations provide concrete solutions, they may not capture the broader spectrum of AI ethics.

A strategic synthesis of existing research with a balance between macro-level ethical guidelines and micro-level investigations of specific risk areas is essential to derive overarching principles and detailed risk assessments. In addition, continued attention to emerging technologies and evolving risk management practices is critical. Comprehensive, in-depth research into potential ethical risks, coupled with adaptability to new technologies, will ensure a nuanced understanding and effective management of evolving AI risks.

This study delves into the potential risks posed by AI events. First, we construct a detailed ontological AI risk model to provide a unified conceptual foundation across multiple scales. Second, we apply this model as a recoding framework to systematically extract and annotate raw news data, resulting in an enriched AI risk event dataset, a real-world repository of AI risk cases enriched with a wide range of ontology-defined attributes. This provides enhanced analytical value. Finally, we conduct a series of analyses, including visual analytics and explainable machine learning, to identify recurring patterns and explore the underlying drivers of AI risk attributes. By integrating ontological modeling with news-based evidence and interpretable AI techniques, this study establishes a scalable, data-driven framework for cross-domain AI risk analysis.

## Background

This background begins by identifying bias and fake news as two critical and highly scrutinized topics within AI ethics. It then examines methodological approaches that integrate macro- and micro-level perspectives to advance the analysis of AI ethics issues. Furthermore, it investigates the role of AI practitioners in the governance of AI-related risks. Finally, it synthesizes prevailing presentation formats in existing literature, delineates the research objectives of this work, and specifies the knowledge gaps it seeks to address.

### Discussion on bias

In studying the issue of how AI causes harm to humans, the predominant focus of risk research is often on specific issues such as bias. Since bias directly threatens the fundamental human value of equality for all, it understandably occupies a prominent place in AI ethics^7^. In the field of bias research in AI systems, researchers have proposed various detection methods and provided specific definitions of bias risk. Arnold et al. claim that bias detection in AI systems can be achieved through experimental verification, highlighting the challenge of hidden risks when information is not disclosed within the AI system scenario^8^. Bias is found in the field of natural language processing (NLP). To address bias in NLP, Sun et al. have presented four measures - modular processing, experimental validation, performance degradation after noise introduction, and prevention of manual processing^9^. They also acknowledge the difficulty of coordinating and generalizing multiple methods in the task of preventing bias risks. Further observations by Aïvodji et al. show that the explanation of the responsibility of black-box effects in AI systems is also susceptible to bias^10^. Different explanatory principles may lead to what appears to be a fair explanation for a black box, but is in fact biased. Taking a broader perspective, they suggest integrating the study of bias with an awareness of the social hierarchies involved in AI development. They advocate for practitioners to establish a collaborative research relationship with community members affected by the development of AI systems, emphasizing the need for mutual feedback mechanisms between practitioners and users^11^. With regard to preventive measures against bias, some researchers argued that relying on technology alone is insufficient. A comprehensive approach involves examining the power relations in the development and use of technology, thereby elevating bias from a technical problem to a broader social issue.

### Discussion on fake news

Attention has also been drawn to the problem of fake news. Zhou et al. and Pan et al. proposed several effective methods for detecting fake news, with one notable approach being the use of knowledge graphs^12,13^. This method departs from the traditional constraints tied to news style and focuses directly on facts as the basis for judgment. This not only increases the accuracy of fake news detection, but also strengthens its credibility. Beyond the technical aspects, Grinberg et al. emphasized the societal impact of fake news, highlighting the amplifying role of social media platforms in its spread^14^. They claim that amplification facilitates the rapid spread of false information, thereby increasing its negative impact on society. Fake news differs from misinformation in that it poses greater risks to social stability and distorts public opinion. As a result, there is an urgent need for more research and action to effectively address this multifaceted problem.

### AI ethics from a micro perspective: specific domain

Applying AI ethical guidelines to a specific domain is challenging. For example, Rajaonah and Zio delved into environmental protection aspect of the ethical AI research^15^. They highlight four main challenges in assessing the potential environmental risks associated with AI. First, environmental protection is complex and interconnected, requiring a comprehensive consideration of various factors. Second, there is a conflict of interest, as different stakeholders may have different positions and interests in environmental protection. Third, resource and financial constraints make it difficult to guarantee the large investments required for effective environmental protection. Finally, the cultural divides in international cooperation can impede collaboration, underscoring the importance of cross-cultural cooperation and understanding in addressing environmental issues. The above challenges show that environmental protection is a very complex issue on which even humans cannot fully agree. How to apply AI ethics in a controversial research field full of various biases and conflicts of interest remains a critical challenge requiring further resolution. This has gradually evolved into a critical topic of studying AI ethics within a specific risk domain.

### AI ethics from a Micao perspective: guidelines

Besides the detailed studies that focus on specific risk aspects of AI ethics, another research direction is committed to constructing a comprehensive guideline. The involved studies operate on a broad scale, examining the risks of AI from different angles. Taeihagh suggested that AI risks arise primarily from the uncertainty and complexity inherent in AI, which pose technological challenges for managers^16^. Another perspective, explored by Garrett et al.^17^, analyzed the educational gap in AI risk perception by examining AI curricula in computer science and identifying missing components such as accessibility, workforce diversity, and sustainability. In the ethical framework of AI, Ulnicane et al. suggested a potential risk stemming from the arms race-like development of AI by institutions worldwide, which could result in lowered ethical standards^18^. Hagendorff synthesized key principles and benefits from different ethical guidelines and introduced a new perspective, emphasizing the need for actionable programs when dealing with AI risks^19^. Waelen explored AI guidelines within a sociological framework from a perspective of critical theory, emphasizing the importance of ethical analysis^20^. Wong et al. examined existing ethical toolkits and found that they tended to focus on individuals rather than social groups^21^. According to Greene et al.^22^, when there is no ethical guidance, the risks associated with AI and machine learning may include discriminatory decision-making, information filtering, manipulation, invasion of privacy, threats to system security, and unemployment. Taddeo and Floridi emphasized the role of artificial oversight in preventing and controlling AI risks and advocated for a robust manual oversight process for effective prevention^23^. In this study of ethical principles embedded in frameworks, researchers illustrated the difficulties of applying different ethical principles through a thought experiment, and found that AI risks are affected by changes in the underlying ethical principles^3–5^. In contrast, Floridi and Cowls proposed a set of universal principles - benefit, do no harm, autonomy, justice, and responsible person - that form the ethical foundation for the AI risk studies in this paper^24^. In addition to the aforementioned studies, Ferretti et al. introduced new research subtopics on AI risks in the big data era, emphasizing the need for AI risk research to adapt to the contemporary development context^25^.

### The role of AI practitioners in risk management

Another distinctive approach to AI risk research focuses on AI practitioners. Morley et al. emphasized the escalating responsibility that comes with the rapid advancement of AI technology^11^. They suggest that AI technicians must not only excel in technical skills, but also cultivate a deep awareness of AI risks. In addition, they advocate for active engagement in society and a vigilant attitude toward evolving laws and policies. Such engagement aims to improve AI practitioners’ understanding of the potential risks associated with AI technologies, and to facilitate better adaptation and compliance with relevant regulations. This enhanced practitioner involvement, in turn, mitigates the negative impact of AI systems on society. This unique research perspective challenges the perception of AI practitioners as mere technology implementers and positions them as bearers of social responsibility. Given that their decisions directly influence the behavior and impact of AI systems, higher professional standards are imperative. This perspective highlights the central role of AI technologists in AI risk management and emphasizes their mission to uphold AI ethics and social responsibility.

### Methodological proposition and research objectives

In order to enhance the practicality of ethical studies, Chen et al. suggested integrating AI ethics guidelines with real-world case data and to construct a knowledge graph for visualization and analysis^6^. They pioneered the concept of a hyper-knowledge graph system that enables visual analysis of AI ethics in legislative, industrial, and corporate contexts.

In summary, existing studies on AI risk have the following limitations:

1. Scope limitation: Research often focuses either on small, specific domains or broad-scale guidelines. In other words, there is a lack of analytical models that bridge the micro and macro perspectives.
2. Lack of detailed and generalizable statistical conclusions: Current studies lack detailed, universally applicable mathematical and statistical insights into AI risk. This makes it difficult for AI practitioners to gain a comprehensive understanding of AI ethics issues from a broader perspective.
3. Insufficient integration of visualization and interpretability analysis: AI risk research lacks approaches that combine visualization with interpretability analysis, resulting in limited insights into the underlying driving factors of AI risks.

To address the above limitations, this study introduces the ontological AI risk model. This model enables both micro-level examination of individual cases and macro-level recoding and analysis of entire datasets, thereby establishing a continuous perspective. Additionally, the model facilitates the derivation of direct conclusions from news data through statistical and visual analysis. Finally, by employing explainable machine learning, the model provides insights into the underlying driving factors of AI risks to a certain extent.

## Methodology

**Fig. 1:**
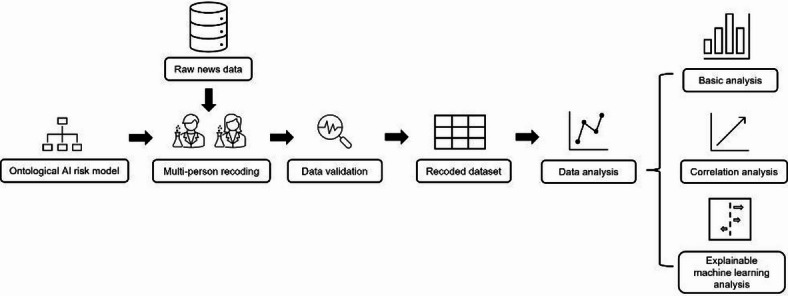
The general workflow diagram.

The research workflow is shown in Fig. 1.The first step is the establishment of the ontological AI risk model. This will be elaborated in detail in the subsequent chapters. Based on the ontological model, multiple researchers used it as a coding guideline to recode the data from the AI incident database^26^. This is due to the limited granularity of attributes in the original AI incident database. Through the recoding process, the database is enhanced with additional attribute annotations. It is worth noting that in the construction of AI incident databases, relevant researchers have conducted rigorous screening procedures to ensure the validity and authenticity of the reported cases. Next, the results of the recoding process are validated and preprocessed to obtain the recoded dataset. The recoded dataset will be used for data analysis, including basic analysis, correlation analysis, and explainable machine learning analysis.

### Ontological AI risk model

**Fig. 2:**
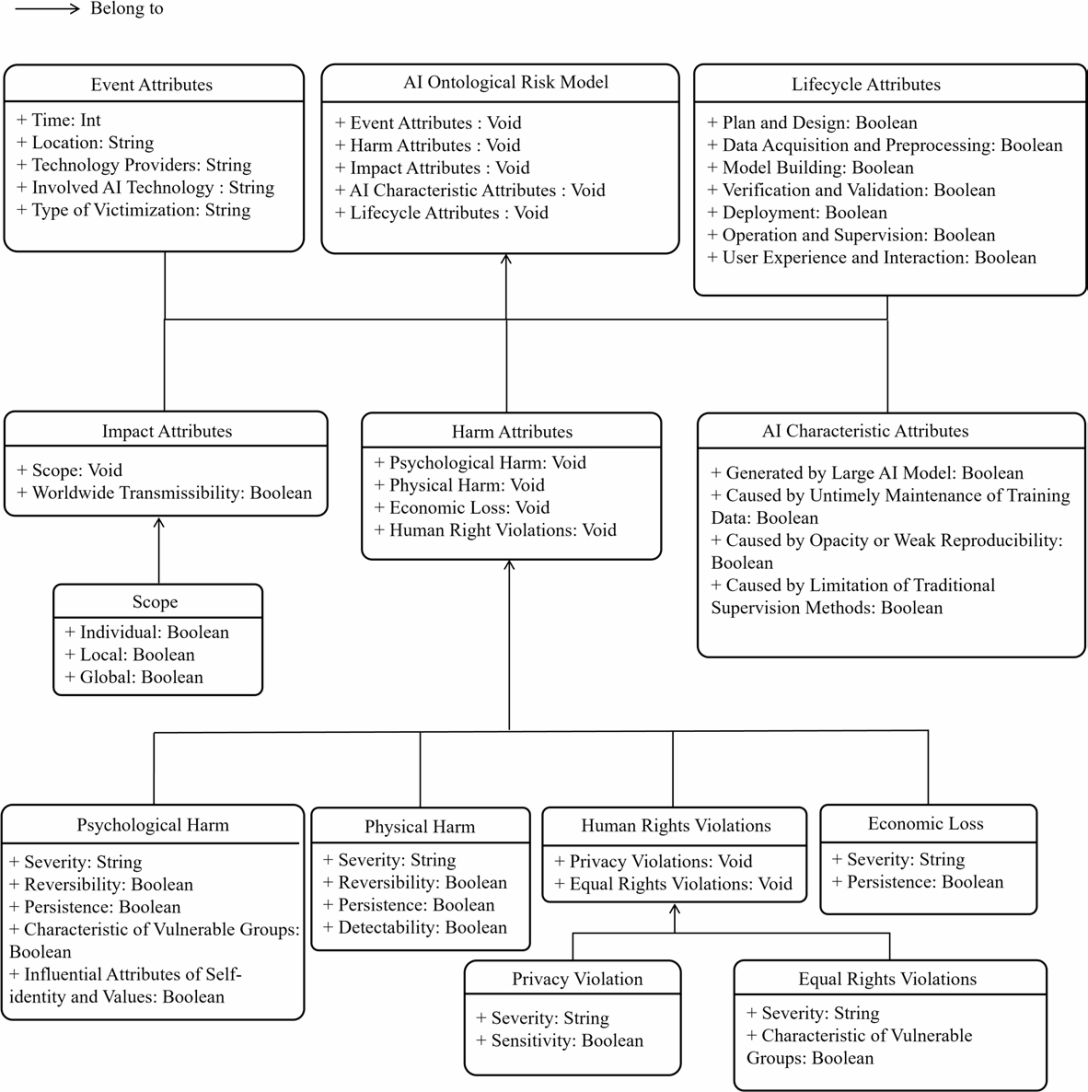
The structure of the ontological AI risk model.

The ontological risk model derived by AI events is demonstrated in Fig. 2. This methodology of ontological modeling to study the ethical issues of AI comes from Chen et al.‘s work on constructing a visual knowledge graph for AI ethics^6^. This model incorporates attributes from five aspects: event, harm, impact, AI characteristics, and position in the lifecycle of data handling.

**Event attributes**: The attribute “**Time**” refers to the exact moment an AI incident occurs, including the start time and duration of the event. The “**Location**” attribute details the geographic location where the AI incident takes place, or the geographic extent of the events if the exact location is unknown. “**Technology Providers**” denote the entities that supply the AI technology involved in the incident. The “**Involved AI Technology**” category refers the types of AI systems implicated in the context of risk. Finally, the “**Type of Victimization**” attribute categorizes the forms of direct victimization resulting from the AI incident.

**Harm attributes**: The severity of harm is categorized into five levels in accordance with recommendations of the German Data Ethics Committee^27^, which is the only detailed categorization of harms caused by AI to humans.

- Level 1: Potential harms with zero or negligible risk, the harms may be classified as Level 1 if the harm is very unlikely to occur in an accident and has a very limited impact on an individual.
- Level 2: Minor harms, minor scratches, abrasions or temporary discomfort may be classified as Level 2, The harms cause some temporary or minor impact on a person’s life, but are usually tolerable.
- Level 3: Conventional harms, the harms that have some degree of ongoing impact on a person’s life and may take some time to recover from. These include some of the more common but very serious injuries, such as moderate burns and broken bones.
- Level 4: Severe harms, the harms have a serious and long-term impact on a person’s life and can require a long period of rehabilitation or permanent disability. For example, severe burns, loss of a limb, or other long-term health problems.
- Level 5: Unbearable harms, the harms that are so severe that the individual cannot tolerate them, potentially resulting in life-threatening or irreversible damage.

Harm attributes provide a detailed framework for understanding the diverse types of harm associated with AI incidents, offering a comprehensive perspective on the potential negative impacts of AI technologies. For Psychological Harm, multiple specific attributes are meticulously defined to capture the complexity of such harm. The attribute “**Severity**” is categorized into one of five levels, indicating the intensity and acuteness of the harm experienced by individuals. “**Reversibility**” measures the extent to which the psychological damage can be undone, emphasizing the potential for recovery and healing. In contrast, “**Persistence**” assesses the duration and enduring nature of the harm, highlighting how long-lasting and possibly permanent the effects can be. The attribute “**Characteristics of Vulnerable Groups**” identifies individuals who are at a disadvantage in social, economic, or cultural contexts, underscoring the disproportionate impact AI incidents may have on marginalized populations. Additionally, “**Influential Attributes of Self-identity and Values**” refer to the profound ways in which psychological harm can disrupt and alter people’s core self-identity and personal values, affecting their sense of self and moral compass.

For Physical Harm, the attribute “**Severity**” is similarly categorized into one of five levels, ensuring consistency with the assessment of psychological harm. “**Reversibility**” measures the extent to which physical harm can be undone, while “Persistence” assesses the duration of the harm. The “**Detectability**” attribute determines the probability of detecting the physical harm caused by the incident, highlighting the challenges in recognizing and addressing such injuries promptly.

Economic Loss attributes include “**Severity**” which is categorized into one of five levels to indicate the financial impact, and “**Persistence**” which assesses how long the economic loss lasts. These attributes help quantify the economic ramifications of AI incidents, providing insight into the financial burdens that may arise.

For Privacy Violation, the attribute “**Severity**” is again categorized into one of five levels, reflecting the intensity of the privacy breach. “**Sensitivity**” measures the degree of privacy sensitivity according to EU standards, indicating the potential impact on personal data and privacy. This highlights the critical importance of safeguarding personal information in the digital age.

In the context of Equal Rights Violation, “**Severity**” is categorized into one of five levels to indicate the impact on equal rights. The “**Characteristics of Vulnerable Groups**” attribute considers whether the violations are specifically targeted at vulnerable groups, emphasizing the necessity of recognizing and addressing the unique challenges faced by these populations. Such consideration helps ensure that AI technologies do not exacerbate existing inequalities or create new forms of discrimination.

**Impact Attributes**: Impact Attributes delineates the various dimensions of impact associated with AI incidents, offering a structured framework for evaluating the extent and spread of these impacts. The scope of impact is categorized into three distinct levels: “**Individual**” “**Local**” and “**Global**”.

Individual scope refers to impacts that are limited to specific individuals, highlighting the personal and direct consequences of AI incidents on single persons.

Local scope describes impacts that affect a localized population, emphasizing the community-level repercussions and regional concerns that arise from AI incidents.

Global scope represents impacts that have widespread ramifications, affecting populations around the world, thus illustrating the far-reaching consequences of certain AI technologies.

The attribute Transmissibility focuses on the potential for these impacts to spread beyond their initial occurrence. Specifically, “**Worldwide Transmissibility**” describes whether risks may have global implications throughout their lifecycle, underscoring the necessity of considering international and cross-border implications. This attribute highlights the importance of global cooperation and regulation to mitigate the pervasive and potentially escalating risks of AI technologies.

**AI characteristic attributes**: With the development of AI, algorithms, services or products that contain AI have demonstrated distinctive characteristics, such as trainability and opaqueness (black-box properties). It is therefore important to ask whether the risk arises from these special characteristics.

Large AI models are typically used for machine learning and deep learning. They work on a large scale with many parameters. They are powerful in various areas, especially natural language processing. The performance of a large AI model partly depends on its training data. If the training data is outdated or no longer reflects reality, the model may fail or produce incorrect results, which can lead to risks. Opaque models are those whose decision-making processes are difficult to understand or explain. This opacity can make it difficult to detect and correct errors or biases in the system. In addition, if the performance of AI systems is not easily reproducible, even if they perform well in one situation, it is difficult to determine whether they will perform well in other situations. This opacity and lack of reproducibility can also lead to risks. In some areas, where AI is replacing non-AI methods with great success, traditional oversight methods may perform poorly if they are not updated.

**Lifecycle attributes**: AI products or services are usually created during the lifecycle of data handling from planning to release^28^. They include the various stages involved in the development and deployment of AI products, providing a structured framework for understanding the comprehensive lifecycle of AI technologies.

**Plan and design**: This stage involves defining the overall goals and architecture of the AI product, with a focus on the goals and scope of the project. It includes defining the problem statement, identifying key performance indicators (KPIs), and developing the overall architecture and functional requirements for the product.

**Data acquisition and preprocessing**: Data are collected and processed for use in training and testing models. This phase encompasses data cleaning, feature engineering, and dataset construction, ensuring that the data are suitable for building robust AI models.

**Model Building**: In this stage, processed data are used to build AI models. This involves selecting appropriate machine learning algorithms, model architectures, and training processes. Model building also requires hyperparameter tuning and validation to ensure optimal performance.

**Verification and validation**: Once the model is built, it undergoes verification and validation. Verification involves examining the specifications, design, and development phases to ensure they meet the intended requirements and specifications. Validation ensures that the final implementation meets the needs and expectations of the end user, including performance evaluation using a validation dataset to check accuracy, robustness, and interpretability. It also includes a review of the model for bias and fairness.

**Deployment**: This phase involves integrating the built and validated AI models into real-world applications. It may include embedding the model into a software system, cloud service, or embedded device to enable the actual automation of a task.

**Operation and supervision**: In this phase, the performance of the model is continuously monitored to identify potential problems or drifts, ensuring the continued validity of the model. It involves monitoring changes in both input data and the output of the model.

**User experience and interaction**: This stage involves evaluating the product’s performance in real-world environments and its impact on users’ experience. It may require regular feedback loops to continually improve the product’s performance and user experience. In the following sections, this attribute is also referred to as “User usage and its influencing factors”.

In Table 1, we provide a detailed introduction to all ontology components, their corresponding categories, and illustrative examples. The ontology model and the detailed definitions of its attributes were designed to establish a rigorous data annotation standard, which guided the recoding process of the dataset.

**Table 1:** Summary table of ontological model.

Component name	Category	Sub-Attributes	Example
Event attributes	Top-level Class	Time, Location, Technology Providers, Involved AI Technology, Type of Victimization	Time = 2022, Location = “Munich”, Technology Providers = “OpenAI”, Involved AI Technology = “Large language model”, Type of Victimization = “students”,
Lifecycle attributes	Top-level Class	Plan and Design, Data Acquisition and preprocessing, Model Building, Verification and Validation, Deployment, Operation and Supervision, User Experience and Interaction	Deployment = True, User Experience and Interaction = False
AI characteristic attributes	Top-level Class	Generated by Large AI Model, Untimely Maintenance of Training Data, Opacity or Weak Reproducibility, Limitation of Traditional Supervision Methods	Generated by Large AI Model = True, Opacity or Weak Reproducibility = False
Impact attributes	Top-level Class	Scope, Worldwide Transmissibility	Transmissibility = True
Scope	Leaf node under Impact	Individual, Local, Global	Global = True
Harm attributes	Top-level Class	Psychological Harm, Physical Harm, Economic Loss, Human Rights Violations	Void
Psychological harm	Leaf node under Harm	Severity, Reversibility, Persistence, Characteristic of Vulnerable Groups, Influence Attributes of Self-identity and Values	Severity = “Level 3”, Reversibility = False
Physical harm	Leaf node under Harm	Severity, Reversibility, Persistence, Detectability	Severity = “Level 3”, Detectability = True
Economic loss	Leaf node under Harm	Severity, Persistence	Severity = “Level 2”, Persistence = True
Human Rights violations	Leaf node under Harm	Privacy Violations, Equal Rights Violations	Void
Privacy violations	Sub-node of Human Rights	Severity, Sensitivity	Severity = “Level 2”, Sensitivity = True
Equal Rights violations	Sub-node of Human Rights	Severity, Characteristic of Vulnerable Groups	Severity = “Level 2”, Characteristic of Vulnerable Groups = True

### Data validation and recoded dataset

In this paper, raw news data were collected from the AI incident dataset^26^. The AI incident database contained 496 events at the start of the experiment. All events were included in this research. These events included in the study are publicly available and span up to the year 2024. Based on this raw dataset and ontological risk model, a new dataset was generated through recoding and prepared for publication. The following section provides a detailed description of the validation metrics used in the manual data recoding process.

**Fig. 3:**
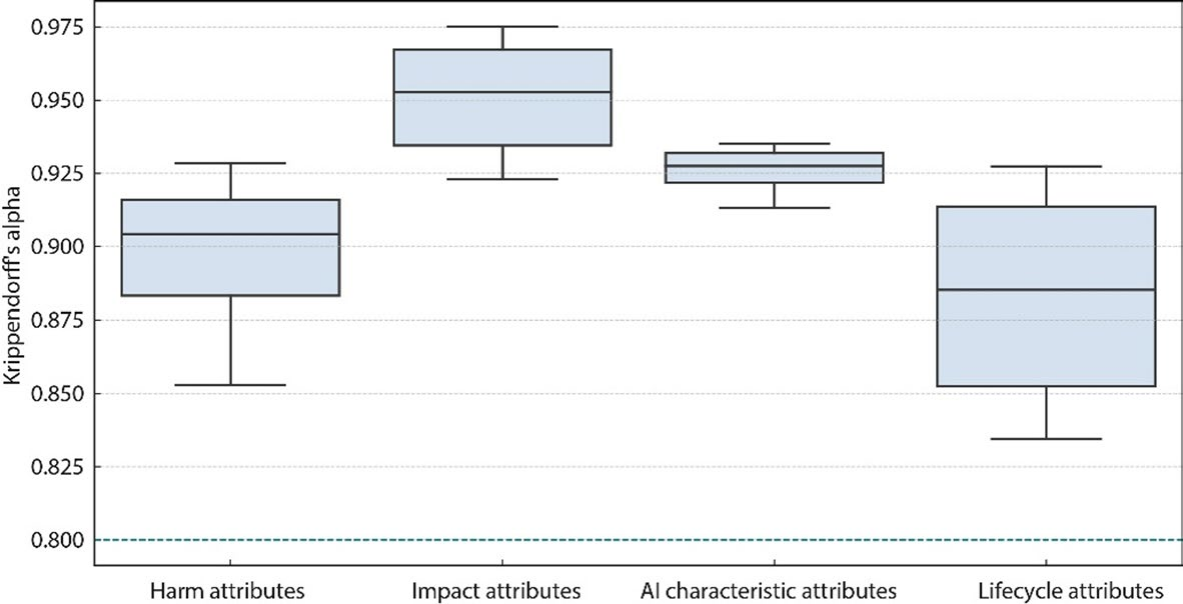
The distribution of Krippendorff’s alpha.

Event attributes are individually labeled due to their objectivity, as they are explicitly described in news data. The principle of labeling other attributes is based on the content analysis theory^29^. According to content analysis theory, data labeling is considered fully reliable when Hayes and Krippendorff’s alpha is greater than 0.800. Data are considered suitable for drawing preliminary conclusions when Hayes and Krippendorff’s alpha is greater than 0.667.

During the coding phase, three coders—each a participant in this research and possessing an academic background in AI ethics—were involved. Prior to individual coding, the coders collectively established a shared understanding of the recoding standards based on the ontological model definitions introduced earlier. Each coder independently labeled the cases to ensure unbiased assessments. To assess the reliability of the coded data, the mean of pairwise Krippendorff’s alpha was then calculated to determine the average agreement. The confidence level of the attributes in this dataset exceeds the standard threshold. Fig. 3 shows the mean Krippendorff’s alpha values for each attribute. For cases where coding discrepancies were identified, all conflicting instances were systematically isolated and re-evaluated. Through a structured discussion anchored in the ontological model, the coders reached a consensus on each disputed case, thereby producing a finalized, harmonized recoded dataset. In the recoded dataset, the attributes are either binary or hierarchical (severity). Events based on natural language can be derived by converting binary and hierarchical data into declarative statements.

A transformed example in the recoded dataset is shown in Table 2. The synopsis of the event is “Google Image returns results that underrepresent women in leadership roles, notably with the first photo of a female “CEO” being a Barbie doll after 11 rows of male CEOs.”

**Table 2:** An example of the event in dataset.

Type of attribute	Content of the example	Recoding process
Event attribute	This record shows that on April 4, 2015, Google, a technology provider, used an AI-powered search algorithm worldwide. The AI technology produced outcomes that reflected sexist bias	The event involves a major global technology provider and the use of an AI-powered search algorithm. The outcome suggests algorithmic sexism, constituting a representative AI-related risk incident
Harm attribute	The psychological harm is conventional harm. It is also reversible and persistent. The victim is in vulnerable groups. It influences self-identity and values. This event may not include physical harm, economic loss and privacy violations. The equal rights violations are conventional harm	The representation of a Barbie doll as the first female CEO—following multiple male CEO images—may perpetuate stereotypes and reinforce gender bias. Such representations can cause psychological harm, particularly to individuals from underrepresented groups, by affecting their self-identity and perceived societal value
Impact attribute	The harm of this event is transmissible. The scope of the event is local	Although originating from a localized platform interaction, the outcome (biased image ranking) is inherently shareable and discussable via screenshots or media coverage. This implies potential transmissibility of harm beyond the initial user
AI characteristic attribute	This event is caused by untimely maintenance of training data	The biased ranking may plausibly result from outdated or imbalanced training data that failed to capture evolving representations of gender in leadership roles. This suggests insufficient updates or oversight in data curation
Lifecycle attributes	The event spans multiple lifecycle stages, including data acquisition and preprocessing, model building, verification and validation, operation and supervision and user experience and interaction stages	The presence of representational bias implicates multiple lifecycle stages: from biased data acquisition and model construction to insufficient validation, lack of post-deployment monitoring, and ultimately the delivery of biased outputs to end-users

### Data analysis

In the data analysis module, we conducted three types of analysis: basic analysis, correlation analysis, and explainable machine learning analysis.

In the basic analysis, we used charts to represent the fundamental statistical information from the ontological AI risk model. Subsequently, in the correlation analysis, we calculated and output the pairwise correlations of the attributes within the ontological AI risk model across the entire dataset.

In the explainable machine learning analysis, we first used the XGBoost model^30^ to perform modeling on five representative attributes: privacy violations, equal rights violations, psychological harm, physical harm, and economic loss. In the machine learning model, the predictor variables were selected from the ontological model by excluding the target variable and all its subordinate branches. For example, in the case of privacy violation, attributes other than privacy violation and its subordinate branches were used as predictor variables in the construction of the machine learning model. The model was built using an XGBoost classifier, with an 80 - 20 split between the training and testing datasets. SHAP (SHapley Additive exPlanations)^31^ was employed to perform interpretability analysis on the trained model. For each model, 5 most important features and their SHAP value (including beeswarm plot) are output. Through this process, the contribution of key attributes and their influence on the target attribute are effectively interpreted for each model.

## Results

### Statistical analysis of various attributes

#### Analysis of event attributes

**Fig. 4:**
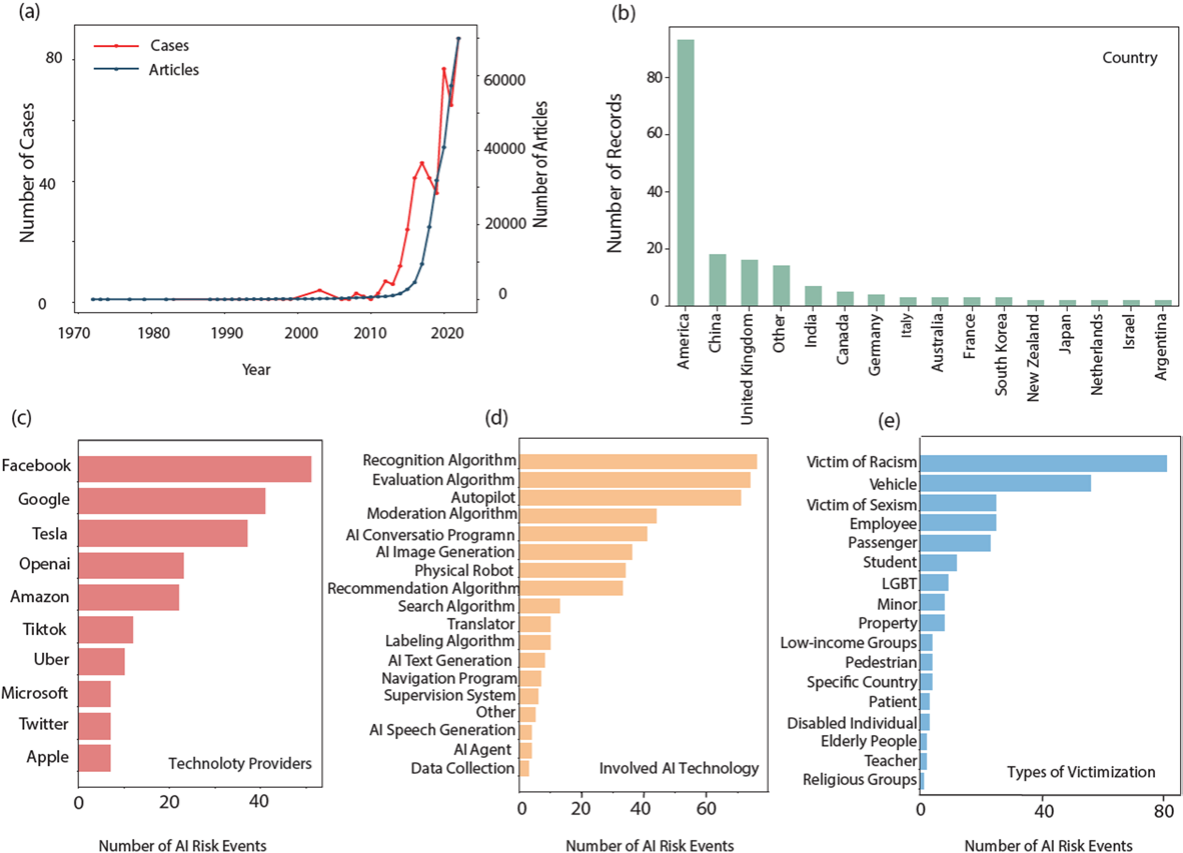
Analysis of event attributes. (a) AI risk events and number of AI-related articles over time. (b) the spatial distribution of AI risk events. (c) the distribution of technology providers of AI risk events. (d) the distribution of involved AI technology of AI risk events. (e) the distribution of victimization type of AI risk events.

The risky AI events were rarely reported before 2000, but they gradually increased from 2000 to 2012, and experienced a spike after 2013. This trend can be attributed to two main factors. First, recent advances in the Internet and mobile communications have brought more relevant events to public attention and expanded the dataset. Second, as the popularity of AI products and services increases, so does the number of risk events and publications. As shown in Fig. 4a, the introduction of deep learning technology, particularly in 2012, marked a significant shift. Subsequent developments have triggered a wave of deep learning research and application, accelerating the overall progress of AI and fueling the rise of AI risk incidents.

The spatial distribution of AI risk is shown in Fig. 4b. Events with identified countries were most prevalent in the United States, followed by China, the United Kingdom and India. This finding may be associated with the prevalence of AI applications in communication platforms such as social media in recent years, as well as globalization trends driven by large AI companies involved in cross-border production and trade.

The distribution of technology providers is shown in Fig. 4c. Among all risk events, the top 5 most involved technology providers were “Facebook”, “Google”, “Tesla”, “OpenAI”, and “Amazon”, accounting for 49.86% of the risk events containing clear providers. There are 17 technology providers involved in the number of times more than twice. These 17 technology providers involved in the incident accounted for 70.03% of the risk events. Fig. 4c shows that AI products from large companies are more widely used and have more reported risks, suggesting that these large companies should take more responsibility for AI risk prevention.

Fig. 4d shows the distribution of involved AI technology of AI risk events. Based on it, the primary areas of AI technology involved were recognition algorithms, evaluation algorithms, autopilot, moderation algorithm and AI conversation programs which together accounted for 63.88%. Among these, identification algorithms, evaluation algorithms, and moderation algorithm carry inherent risks due to their susceptibility to human interference during redesign. As these algorithms are mature and widely used, they require increased security attention. On the other hand, autonomous driving and AI conversational programs, while posing more challenges due to their nascent nature, have significant future potential. Therefore, ensuring the security of these technologies is critical to prevent potential risks.

Fig. 4e represents the distribution of victimization type of AI risk events. The percentage of total risk events with a clear victimization type is 52.40%, with the most events involving discrimination or victimization of racially profiled people, vehicles, staff, people subjected to gender discrimination, and students with a total of 79.68%.

#### Harm of AI risk

**Fig. 5:**
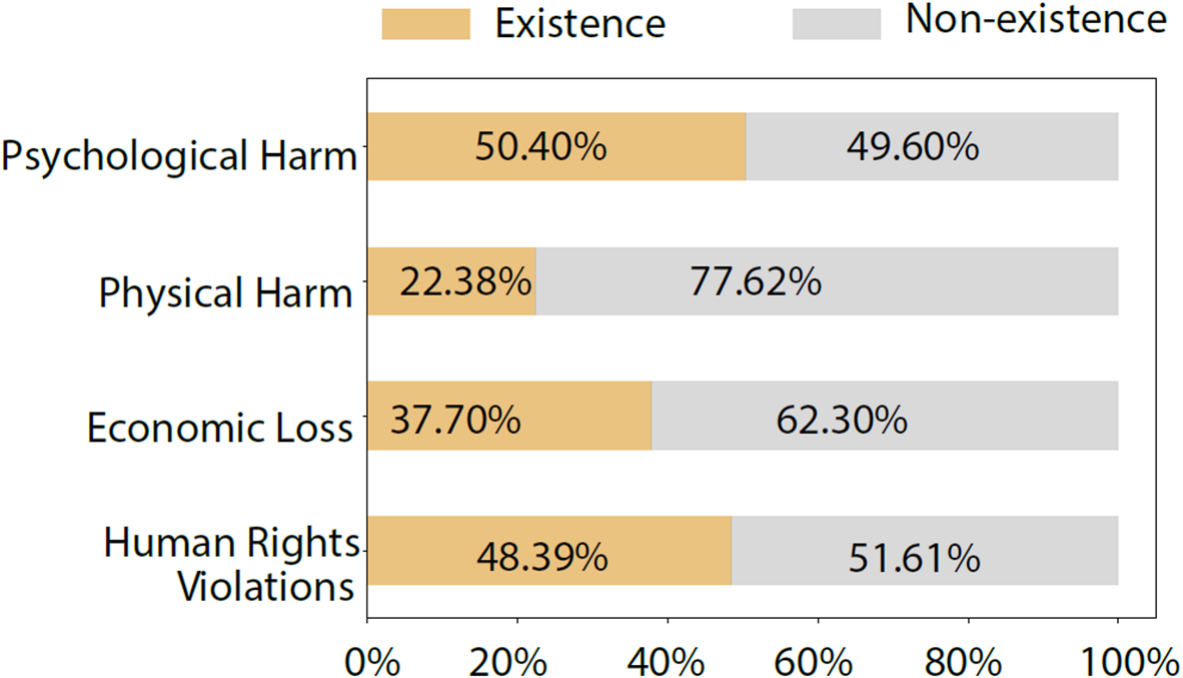
The percentage of the four types of harm in AI risk events.

The percentage of the four types of harm in AI risk events is shown in Fig. 5. 48.3% of all events involved human rights violations. Psychological harm was involved in 50.91% of risk events, physical harm in 22.38%, and economic harm in 37.70%.

**Fig. 6:**
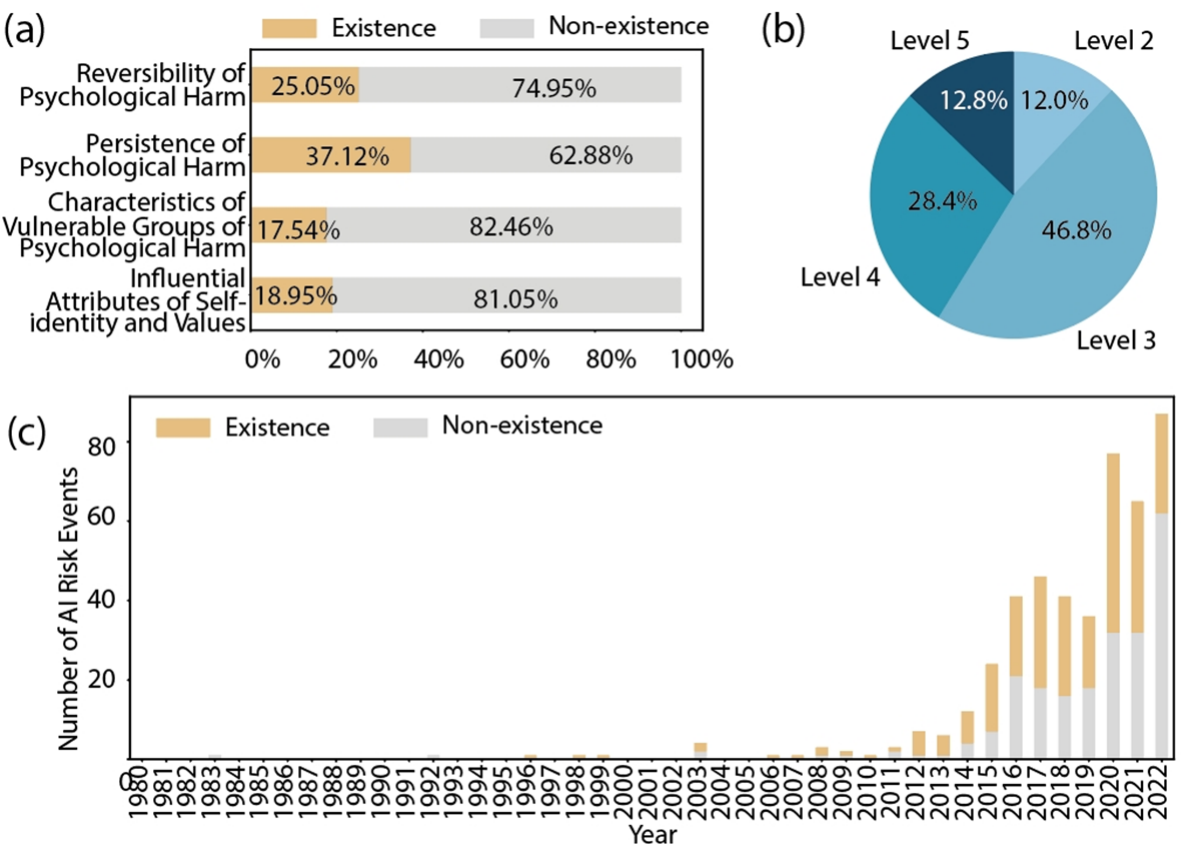
The visual overview of AI risk events with psychological harm. (a) the proportions of AI characteristic attributes. (b) the severity distribution. (c) the fluctuations over time in the percentage of psychological harm.

Fig. 6a demonstrates that among all risk events with psychological harm, 17.54% involve vulnerable groups. 25.05% are reversible. 18.95% affect self-identity and values. 37.12% result permanent harm. It is evident that a significant proportion of risk events resulting in psychological harm involve vulnerable groups, suggesting an urgent need for targeted interventions to protect these populations. In addition, the high proportion of reversible harm events suggests the potential effectiveness of mitigation strategies in addressing such risks. However, the significant proportion of risk events that cause permanent harm underlines the importance of implementing robust preventive measures. Fig. 6b shows severity levels, with level 2 accounting for 12.0%, level 3 for 46.8%, level 4 for 28.0% and level 5 for 12.8%. Fig. 6c shows a concerning trend of increasing annual occurrences of psychological harm events, albeit with a decreasing proportion relative to all types of harm. This suggests a potential improvement in overall risk management strategies, although continued vigilance and proactive measures are required to address the evolving landscape of AI-related risks effectively.

**Fig. 7:**
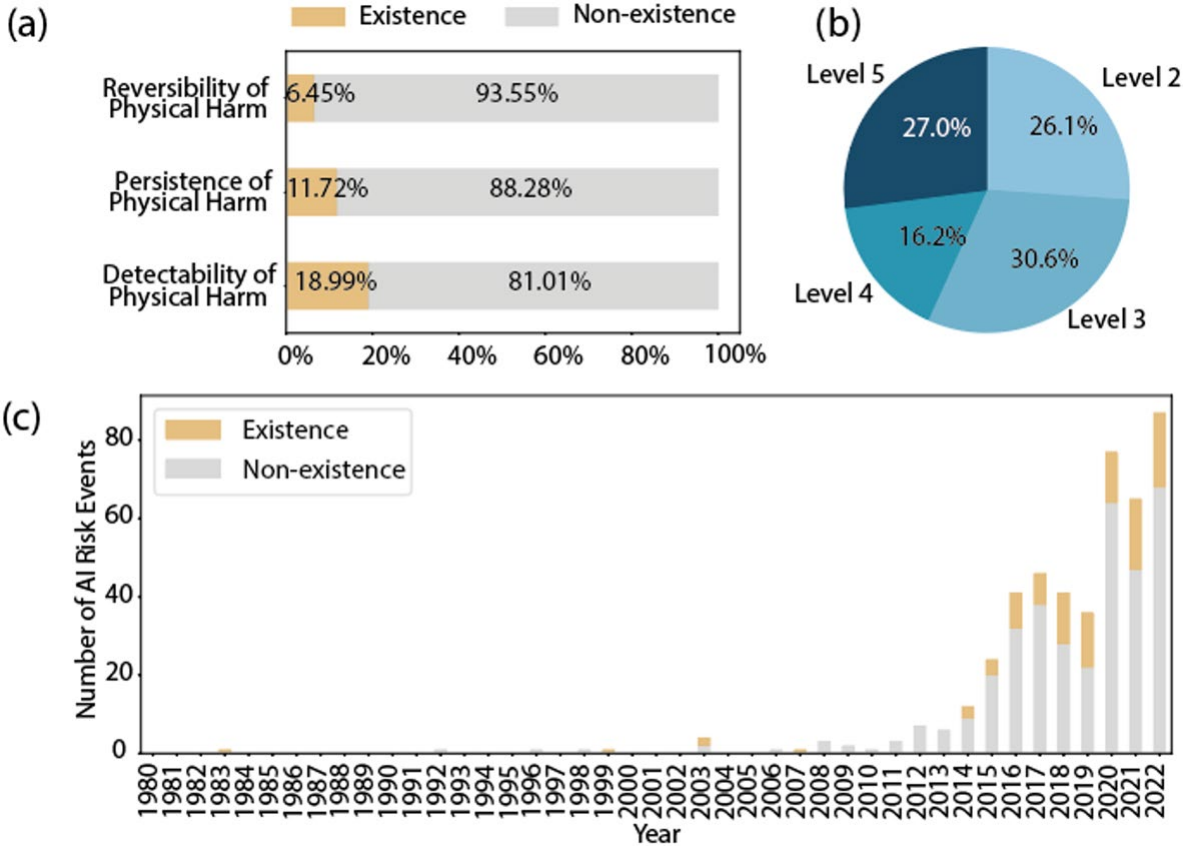
The visual overview of AI risk events with physical harm. (a) the proportions of AI characteristic attributes. (b) the severity distribution of physical harm. (c) the fluctuations over time in the percentage of physical harm.

For risk events involving physical harm as shown in Fig. 7a, 6.45% are reversible. 18.99% are detectable, and 11.72% cause permanent physical harm. A considerable percentage of these events are reversible, suggesting the potential effectiveness of intervention strategies. In addition, the detectability of a significant proportion of physical harm events implies opportunities for early detection and prevention. However, the presence of events resulting in permanent physical harm underscores the urgency of implementing rigorous preventive measures.

With regard to the severity of physiological harm as shown in Fig. 7b, the severity in level 2 accounts for 26.1%, level 3 for 30.6%, level 4 for 16.2%, and level 5 for 27.0%. As can be seen from Fig. 7c, although the number of events with physical harm fluctuates significantly over time, the overall proportion decreases. This indicates potential improvements in risk mitigation efforts, although continued vigilance and proactive measures are essential to effectively address emerging challenges.

**Fig. 8:**
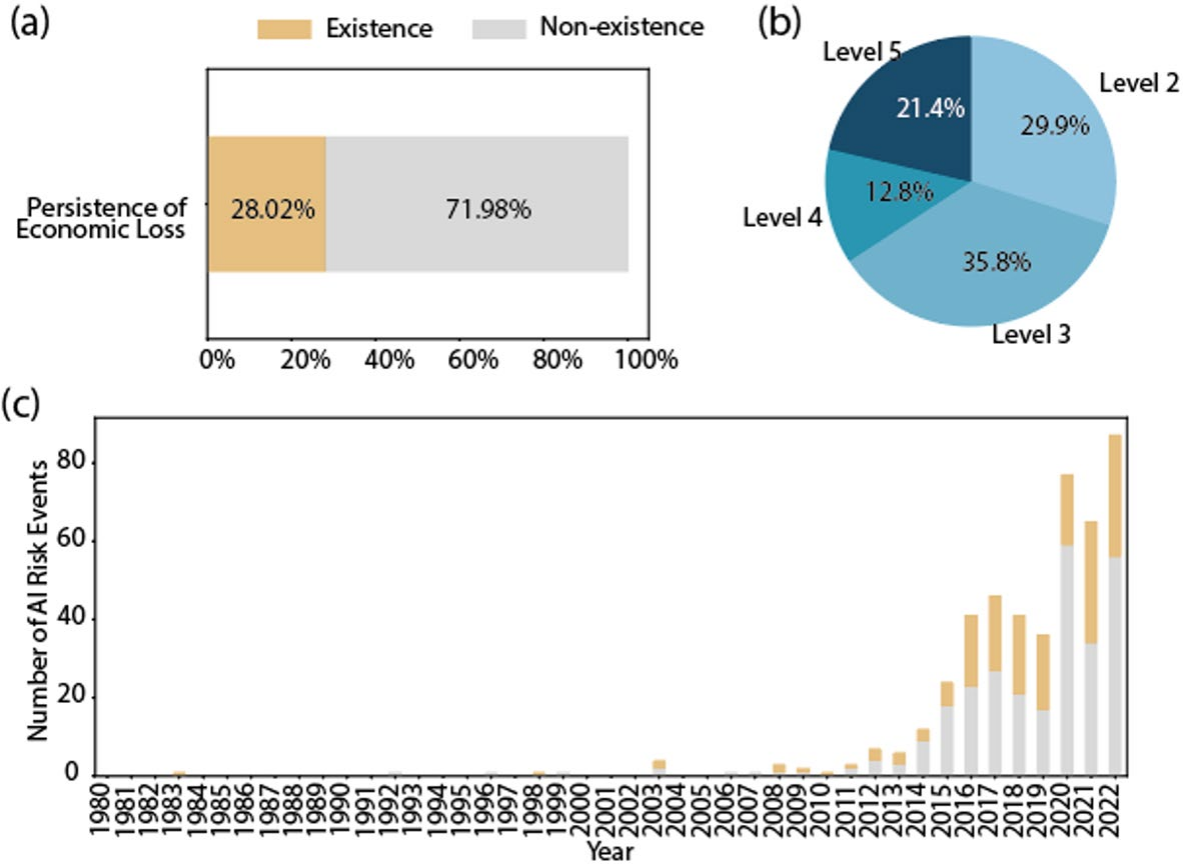
The visual overview of AI events with economic loss. (a) the proportions of persistence of economic loss events. (b) the severity distribution of economic loss. (c) the fluctuation in the percentage of economic loss events over time.

According to Fig. 8a, 28.02% of risk events with economic loss contain persistent loss, highlighting the enduring impact on affected entities. In Fig. 8b, the severity of economic loss is categorized as level 2 in 29.9% of economic loss risk events, level 3 in 35.8%, level 4 in 12.8% and level 5 in 21.4%. According to Fig. 8c, the percentage of risk events with economic loss has remained relatively stable over time at approximately 40%. This indicates an ongoing challenge in mitigating the risk of economic loss and the need for sustained efforts to address underlying vulnerabilities. Overall, these findings underscore the importance of implementing effective risk management strategies to minimize the economic impact of such events on affected companies and the broader ecosystem.

**Fig. 9:**
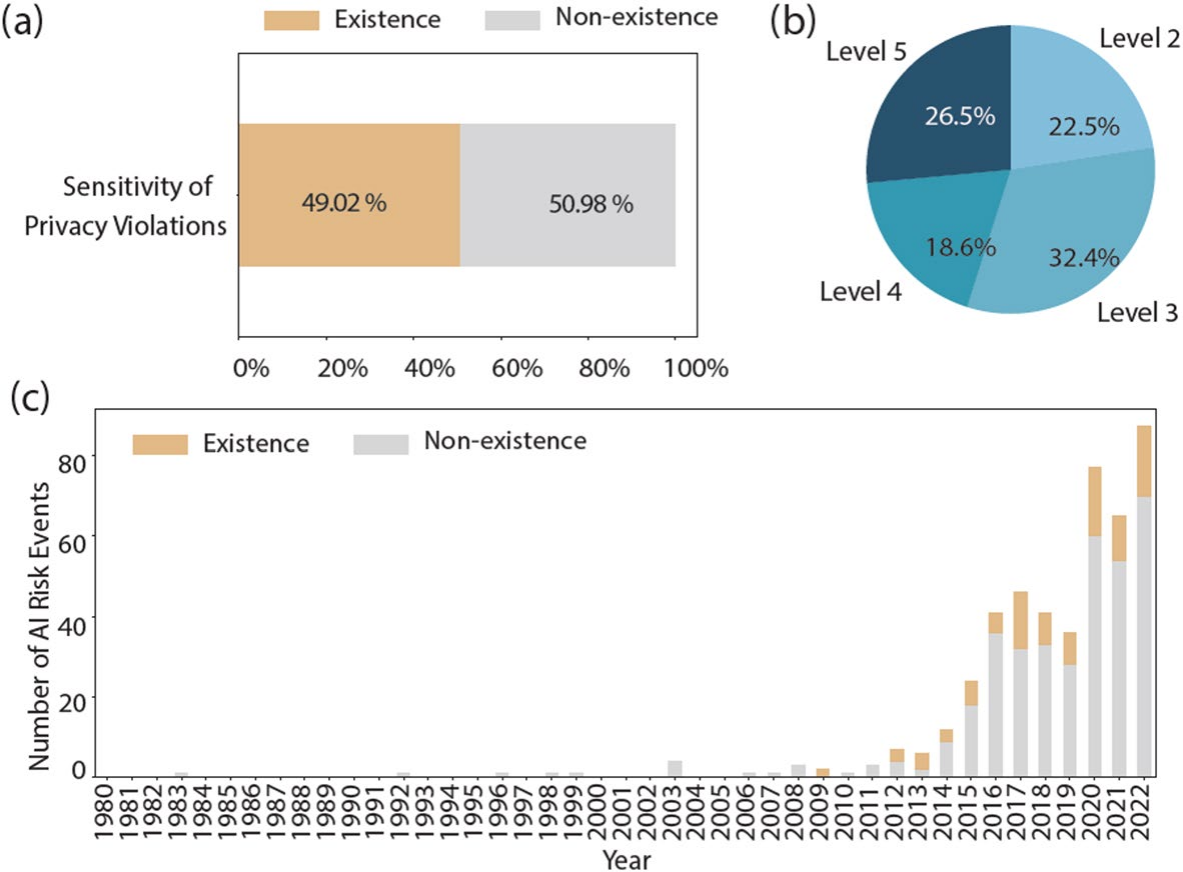
The visual overview of AI events with privacy violations. (a) the proportions of characteristic attributes of privacy violations. (b) the severity distribution of privacy violations. (c) the fluctuation in the percentage of privacy violations events over time.

human rights violations are categorized as privacy violations and equal rights violations.

Among the risk events involving privacy violations shown in Fig. 9a, 49.02% of them involved sensitive privacy. That is, nearly half of these events involve sensitive data breaches, indicating the severity of the compromised information. In Fig. 9b, the severity of privacy violation is categorized as level 2 in 22.5%, level 3 in 32.4%, level 4 in 18.6%, and level 5 in 26.5%. According to Fig. 9c, risk events involving privacy violations are consistently around 20% over time. This persistent level underscores the ongoing challenge of protecting privacy in the face of evolving threats and technologies. Overall, these findings underscore the critical importance of robust privacy policies and regulatory frameworks to mitigate the risks of data breaches and to uphold individuals’ rights to privacy and data protection.

**Fig. 10:**
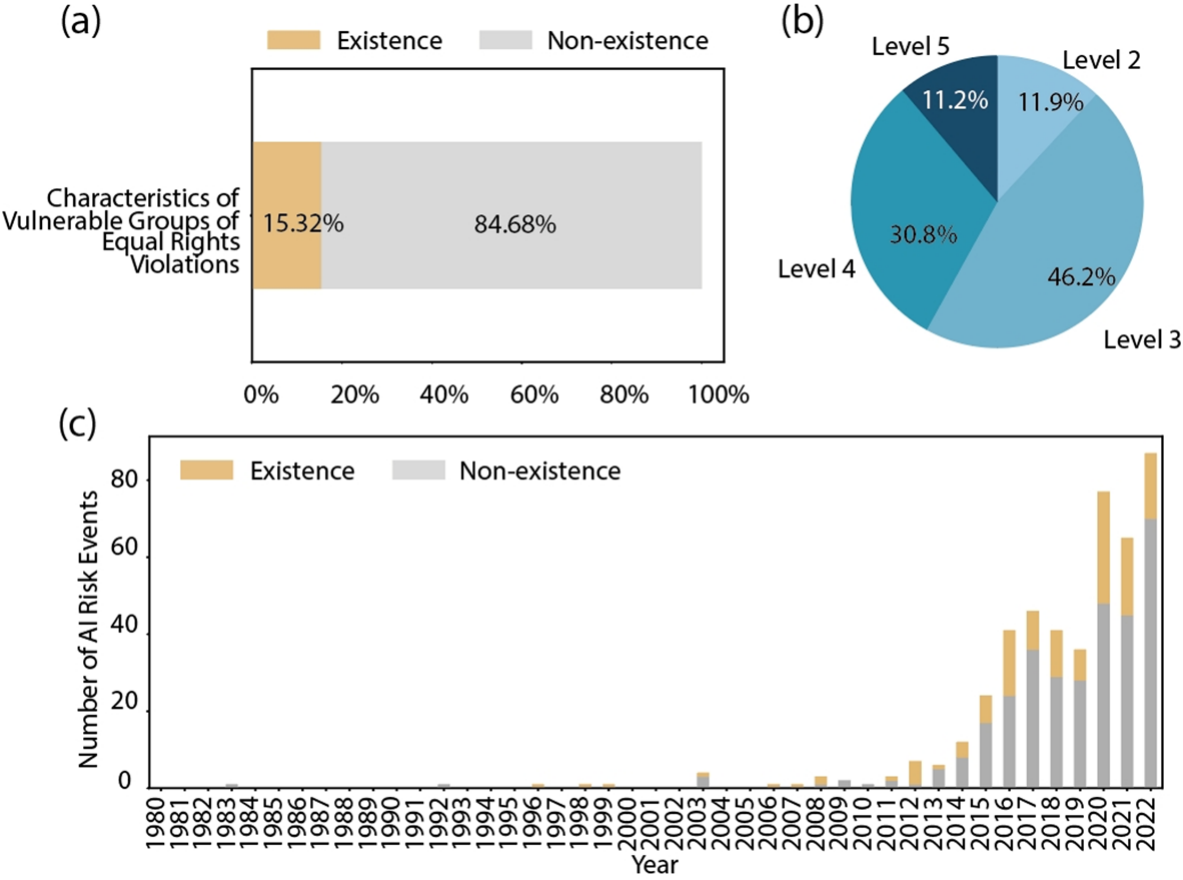
The visual overview of equal rights violations. (a) the proportions of characteristic attributes of equal rights violations. (b) the severity distribution of equal rights violations. (c) the fluctuation in the percentage of equal rights violations events over time.

Among the risk events involving equal rights violations shown in Fig. 10a, 15.32% contain vulnerable groups in terms of equal rights. In Fig. 10b, the severity of equal rights violations is categorized as level 2 in 11.9%, level 3 in 46.2%, level 4 in 30.8%, and level 5 in 11.2%. According to Fig. 10c, risk events involving equal rights violations consistently accounted for approximately 30% of total risk events. This persistent level underscores the ongoing challenge of addressing systemic inequalities and upholding principles of equality and justice. Overall, these findings highlight the critical need for comprehensive measures and policies to prevent and address equality violations, promote inclusion and ensure equitable treatment for all individuals and communities.

#### Impact attributes

**Fig. 11:**
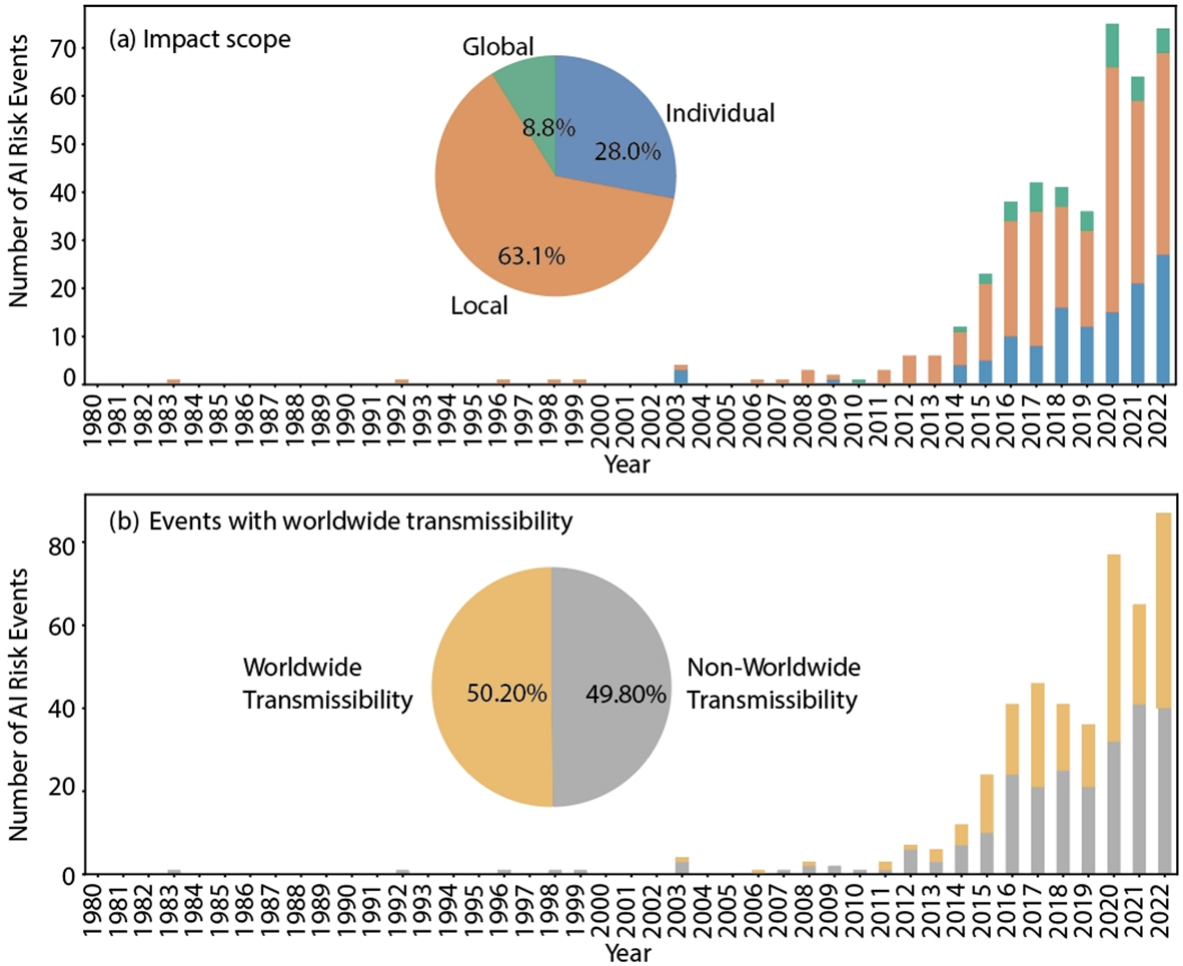
(a) the percentage of events with 3 different impact scopes over time. (b) the percentage of events with worldwide transmissibility over time.

According to Fig. 11, in terms of impact scope of AI risk events, 6.5% of the events had an undefined scope. Of the risk events with a defined scope, 8.8% are global, 28% are individual, and 63.1% are local. Risk events affecting individuals and localized populations have increased significantly from in number, although their percentage of the total has remained relatively stable over time. This suggests a consistent pattern of localized impacts despite an overall increase in the number of AI risk events. These findings underscore the need for targeted interventions and tailored risk management strategies to address the specific needs and vulnerabilities of affected individuals and communities. They also highlight the importance of understanding the localized nature of many AI-related risks and the potential implications for policy development and implementation. Overall, these findings enhance our understanding of how AI risk events are distributed and evolve over time, thereby supporting more effective mitigation strategies and policy planning.

Fig. 11 reveals significant insights into the transmission potential of AI risk events. Just over half, 50.20%, have the capacity for worldwide dissemination, facilitated by the pervasive influence of social media platforms. This highlights the interconnected nature of global technological ecosystems and the role of social media in amplifying the spread of AI risks. Moreover, the persistent proportion of risk events with global transmission capabilities, which remains consistently around 50% after 2015, underlines the ongoing challenge of mitigating and controlling such risks. Effective prevention and control strategies should therefore incorporate measures to enhance social media governance and AI-related products and services provided by globally influential corporations. By addressing both the technological and social dimensions of AI risk transmission, stakeholders can work towards mitigating the potential harms associated with global dissemination and promoting responsible AI development and deployment. Overall, these findings reveal the need for collaborative efforts across sectors to address the complex challenges posed by the global transmission of AI risks and ensure the responsible and ethical advancement of AI technologies.

#### AI characteristic attributes

**Fig. 12:**
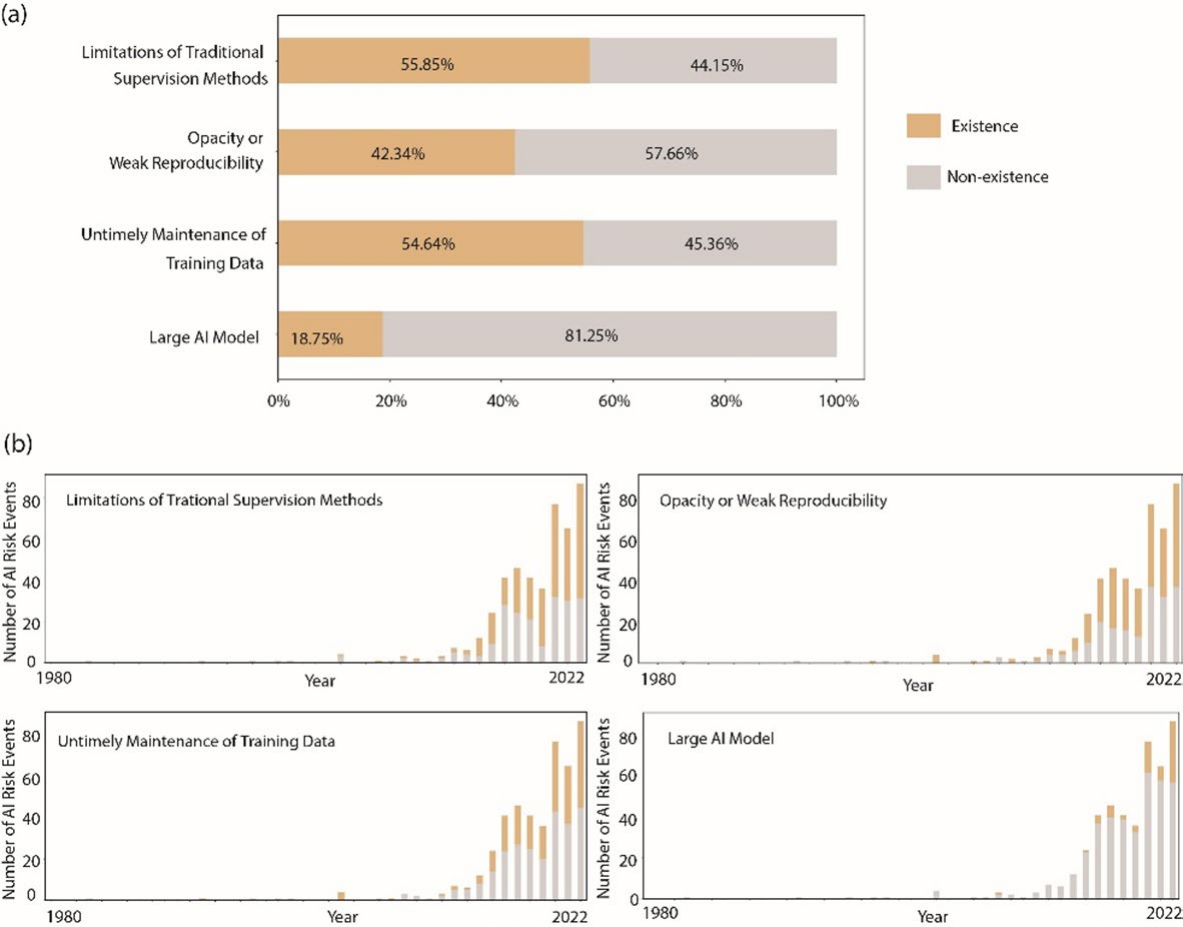
(a) The proportion of risk events with four AI characteristic attributes. (b) the fluctuations in the percentages of events with individual AI characteristic attributes.

Fig. 12 provides critical insights into the factors contributing to AI risk events and their trends over time. Among the various reasons identified, the limitations of traditional oversight methods emerge as the predominant cause, accounting for 55.85% of risk events. This highlights the inadequacy of existing regulatory frameworks and oversight mechanisms to effectively manage AI-related risks. In addition, untimely maintenance of training data emerges as another significant contributor, accounting for 54.64% of risk events. This underscores the importance of ongoing data quality assurance and maintenance practices in ensuring the reliability and integrity of AI systems. Opacity or poor reproducibility is also a significant factor, contributing to 42.34% of risk events. The increasing prevalence of risk events attributed to large AI models (18.75% of the total) shows the unique challenges posed by the complexity and scale of such systems.

The trends observed in these risk factors reveal notable patterns. Risk events involving large models have shown a gradual increase in recent years, reflecting the growing adoption and complexity of AI technologies. Similarly, the percentage of risk events attributed to untimely maintenance of training data has consistently hovered around 50%, indicating an ongoing challenge in data management practices. In addition, the percentage of risk events related to opacity or weak reproducibility is steadily increasing year over year, highlighting the need for greater transparency and accountability in AI development and deployment processes. Of particular concern is the escalating trend of risk events resulting from the limitations of traditional oversight methods, which have surpassed the 50% threshold in recent years. This underscores the urgency of enhancing regulatory frameworks and oversight mechanisms to address effectively the evolving landscape of AI-related risks.

These findings show the need for comprehensive risk management strategies that encompass regulatory, technical, and ethical dimensions. By addressing the root causes identified in Fig. 12 and adapting to emerging trends, stakeholders can work to promote the responsible and ethical development and deployment of AI technologies.

#### Lifecycle attributes

**Fig. 13:**
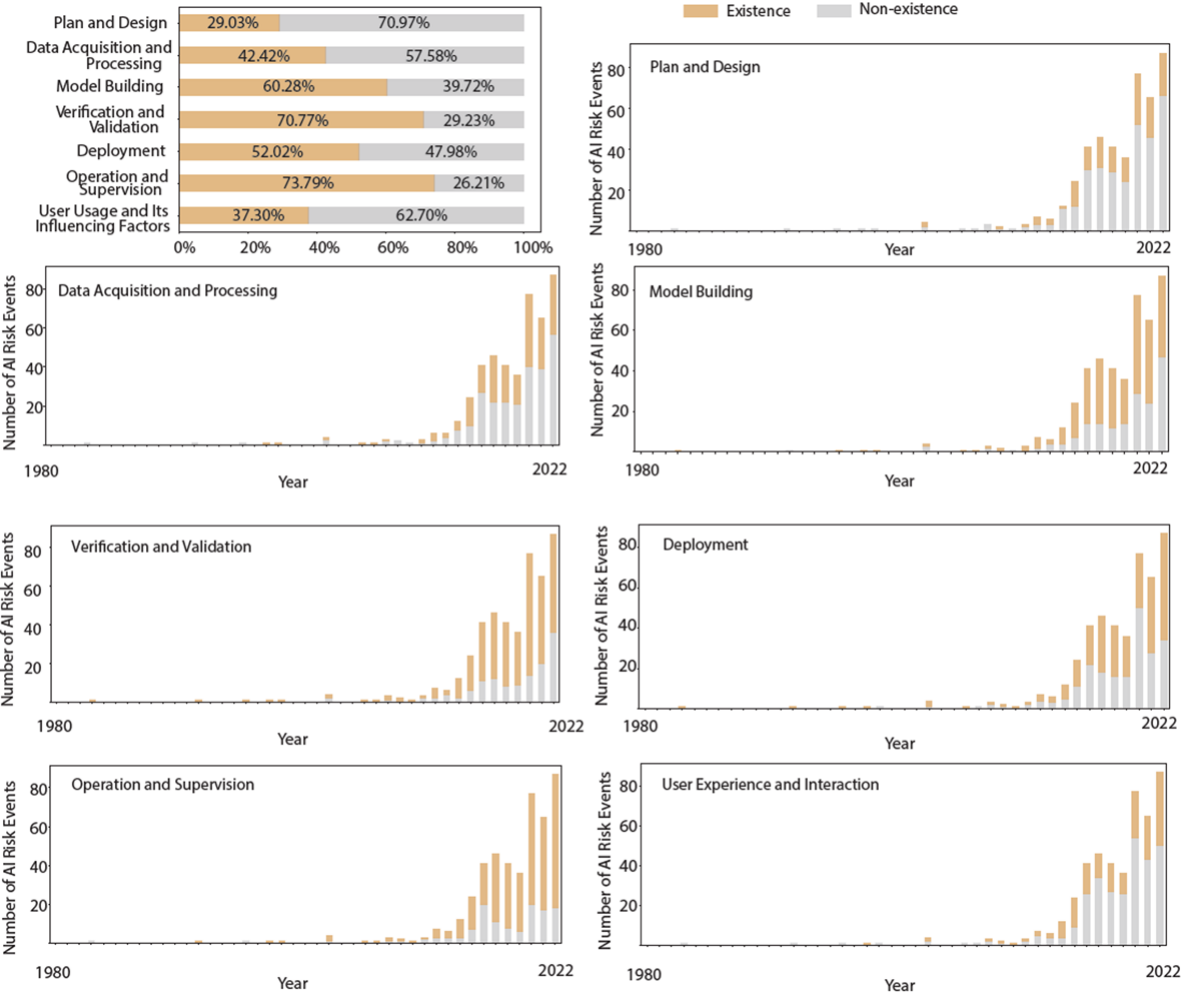
(a) The distribution of risk events in AI product lifecycle. (b) the fluctuations in the percentages of events in various stages on the lifecycle.

Fig. 13 shows the distribution of stages in AI product lifecycle where these events occur can be categorized as 29.03% in plan and design stage, 42.42% in data acquisition and preprocessing stage, 60.28% in model building stage, 70.77% in verification and validation, 52.02% in deployment, 73.79% in operation and supervision and 37.30% in user usage and its influencing factors (corresponding to the “user experience and interaction” stage defined in the ontological model).

Fig. 13 provides valuable insight into the distribution of AI risk events across the different stages of the product lifecycle. The largest proportion of risk events, 73.79% of the total, occur during the operation and monitoring phase, indicating the ongoing challenges of monitoring and managing AI systems in real-world environments. Because operational environments may introduce unpredictable user behaviors, dynamic data shifts, and evolving threats, flaws not evident during controlled testing could emerge post-deployment. The complexity of real-world ecosystems likely amplifies risks through increased pathways for errors and vulnerabilities. In contrast, model-building occurs under more stable and controlled conditions, where issues are more easily detected internally. This disparity highlights the need for robust post-deployment governance and adaptive risk management. The second largest proportion, 70.77% of the total, occurs during the verification and validation phase. This highlights the critical importance of rigorous testing and validation processes to identify and mitigate potential risks prior to deployment. Additionally, 60.28% of events occurred during the model building phase, highlighting the need for careful consideration of algorithmic design and training processes to mitigate the risks associated with model biases and errors. Similarly, risk events during the deployment phase, accounting for 52.02% of the total, underscore the importance of robust deployment practices to ensure seamless integration and functionality of AI systems in production environments.

The percentage of risk events occurring in plan and design stage is decreasing year by year. The percentage of risk events occurring in data acquisition and preprocessing stage increase slightly in recent years, to just over 50%. The percentage of risk events occurring in model building stage is consistently around 70%. The percentage of risk events occurring in verification and validation stage is consistently around 80%. The percentage of risk events occurring in deployment stage is consistently around 50%. The proportion of risk events occurring in operation and supervision stage is increasing. The proportion of risk events occurring in user usage stage is consistently around 30%. The distribution of risk events across phases shows certain trends over time. While the percentage of risk events in the planning and design stage has decreased over time, the percentage of events in the data collection and preprocessing stage has increased slightly in recent years, exceeding 50%. This suggests a shift in focus toward addressing challenges related to data quality and preprocessing techniques. In addition, the consistency in the percentage of risk events during the model building and verification/validation phases highlights the ongoing challenges and complexities associated with these phases of the AI product lifecycle.

Overall, these results suggest that AI risk management requires comprehensive strategies that span the entire product lifecycle. By identifying and addressing risks at each stage, stakeholders can work to improve the safety, reliability, and trustworthiness of AI systems.

### Correlation analysis of attributes

**Fig. 14:**
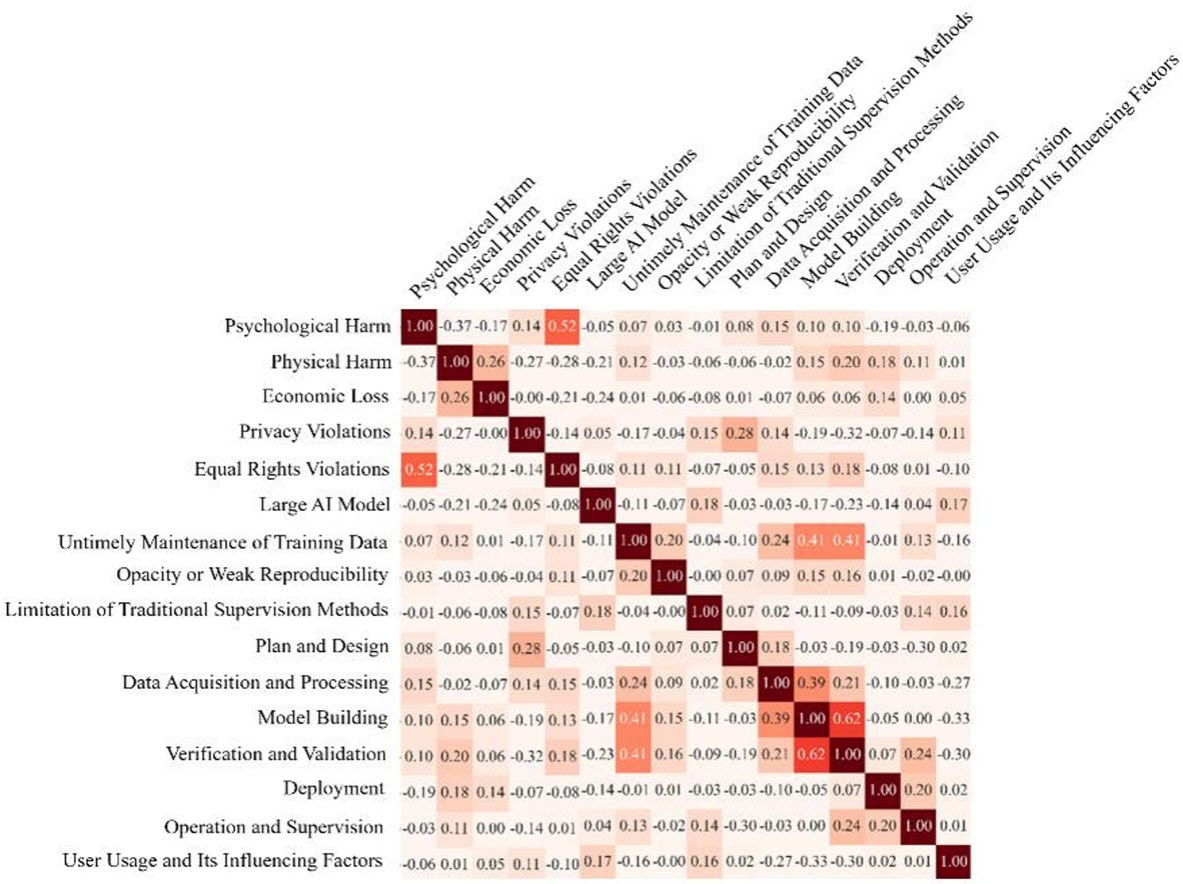
The correlation matrix of attributes.

When investigating AI risk events, distinctions are made based on categorical attributes such as technology provider, AI technology, and type of victimization. These attributes are interrelated and jointly shape the patterns and distribution of AI risk events. Based on the cases collected in this research, the correlations between the attributes were analyzed and some preliminary interpretive insights were drawn to better understand these relationships. Fig. 14 shows a correlation matrix of harm attributes, AI characteristic attributes and lifecycle attributes. The correlation between “Model Building” and “Verification and Validation” is 0.62, suggesting that there is a strong correlation between these attributes. This association is indicative of the interdependence between the quality of the constructed model and the efficacy of the verification and validation processes. A meticulously crafted model typically undergoes testing and validation to ascertain its accuracy and reliability. Conversely, shortcomings or deficiencies in the model-building phase may surface during verification and validation, establishing a correlation between these attributes.

The correlation between “Model Building” and “Untimely Maintenance of Training Data” is 0.41, indicating that there is a moderate positive correlation. This is likely attributed to the consequential effect of timely maintenance of training data on the process of model building. Delays or lapses in updating and maintaining the training data utilized for model construction can impact the quality and precision of the resultant models. Thus, the correlation suggests that challenges in training data maintenance may influence the overall model building process.

The correlation between “Psychological Harm” and “Equal Rights Violations” is 0.52, indicating a significant relationship. This correlation is likely explained by the psychological consequences of violations of equal rights. Instances of unequal treatment or discrimination can result in psychological harm, such as distress, anxiety, or other negative emotions. Therefore, the correlation underscores the connection between individuals’ psychological well-being and the occurrence of equal rights violations, suggesting a cause-and-effect relationship.

### Analysis based on explainable machine learning

**Fig. 15:**
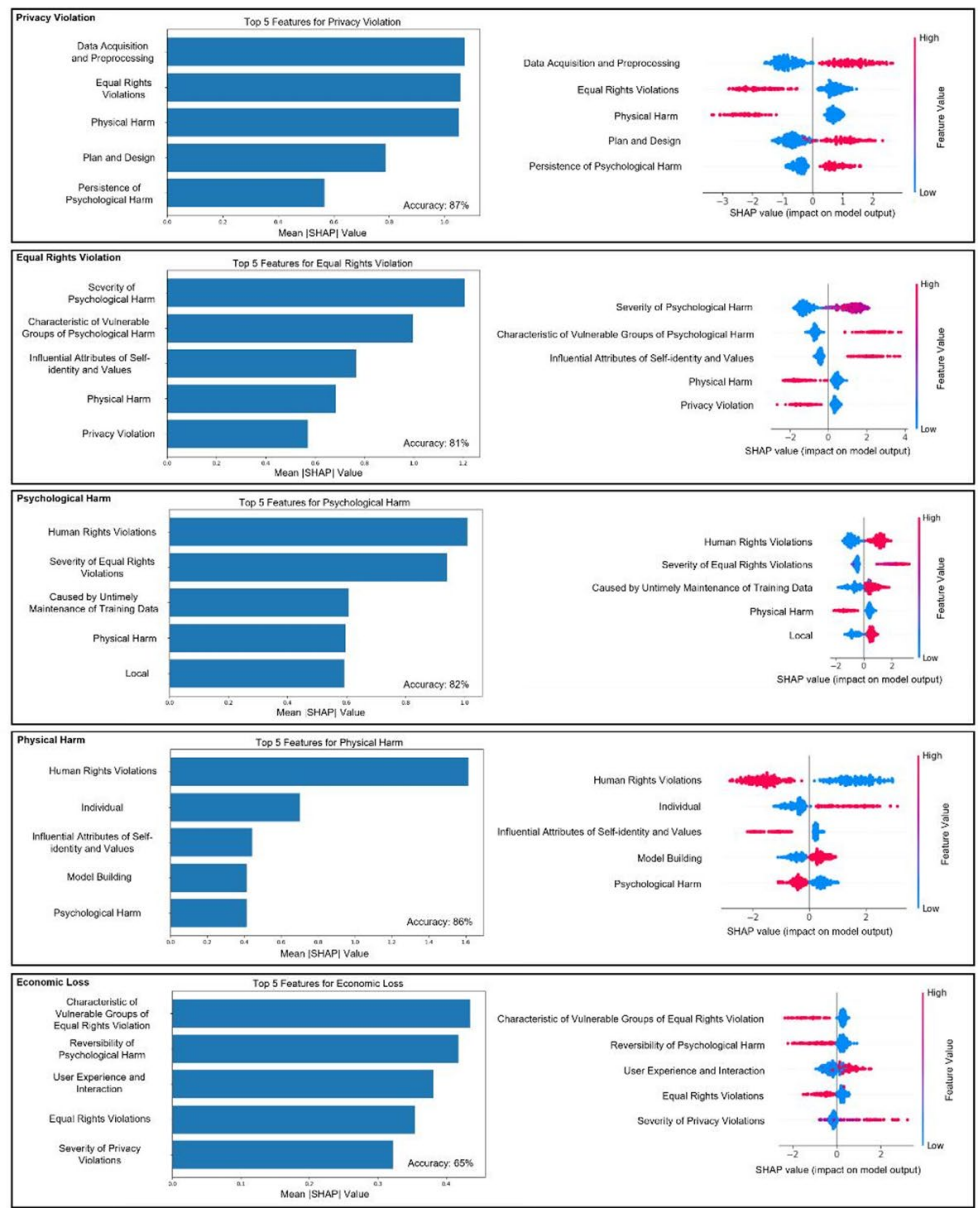
The result of analysis based on explainable machine learning.

Using datasets from the ontological risk model, we applied XGBoost with SHAP for interpretability analysis on five attributes: privacy violations, equal rights violations, psychological harm, physical harm, and economic loss. Fig. 15 shows the top five features and their SHAP values. In Table 3, we present the evaluation metrics of the five models.

**Table 3:** XGBoost model evaluation Metrics.

Category	Accuracy	Precision	Recall	F1 Score
Privacy violation	0.8700	0.8671	0.8700	0.8684
Equitable Rights violation	0.8100	0.8176	0.8100	0.8130
Psychological harm	0.8182	0.8185	0.8182	0.8180
Physical harm	0.8600	0.8550	0.8600	0.8557
Economic loss	0.6500	0.6413	0.6500	0.6440

For the results of privacy violation, the top five important features of the privacy violation prediction model and their mean SHAP values were first presented. Ranked by importance, Data Acquisition and Preprocessing emerged as the most significant feature, exerting the greatest influence on the model output. Then it was followed by Equal Rights Violations, Physical Harm, Plan and Design, and Persistence of Psychological Harm. Furthermore, for Data Acquisition and Preprocessing, Plan and Design, and Persistence of Psychological Harm, higher feature values corresponded to positive SHAP values. This indicates that issues in these areas are significant driving factors behind privacy violations, especially Data Acquisition and Preprocessing, showing its critical role in privacy breaches.

In the prediction model for Equal Rights Violations, the top five most important features and their mean SHAP values were extracted. Ranked by importance, they are: Severity of Psychological Harm, Characteristics of Vulnerable Groups of Psychological Harm, Influential Attributes of Self-identity and Values, Physical Harm, and Privacy Violation. Among these, Severity of Psychological Harm stands out as the most significant feature influencing the model output, highlighting its central role in predicting equal rights violations. For Severity of Psychological Harm, Characteristics of Vulnerable Groups of Psychological Harm, and Influential Attributes of Self-identity and Values, higher feature values correspond to positive SHAP values. This indicates that the presence of these features (with higher values) significantly drives the model’s prediction of equal rights violations.

In the Psychological Harm prediction model, five most influential features, ranked by their mean SHAP values, were identified as follows: Human Rights Violations, Severity of Equal Rights Violations, Caused by Untimely Maintenance of Training Data, Physical Harm, and Local Factors. Among these, Human Rights Violations stands out as the most impactful feature, with the highest mean SHAP value, emphasizing its critical role in shaping the model’s predictions. Both Human Rights Violations and Severity of Equal Rights Violations emerged as the most impactful features in the model, indicating that human rights and equal rights issues are key drivers of psychological harm. The feature Caused by Untimely Maintenance of Training Data highlights that risks associated with outdated training data can lead to psychological harm. Possibly, untimely maintenance of training data can cause AI systems to produce biased, misleading, or inappropriate outputs, which can erode users’ trust, provoke emotional distress, or reinforce harmful stereotypes, thereby inflicting psychological harm. Although Physical Harm and Local Factors have relatively lower mean SHAP values, they still contribute meaningfully to the prediction of psychological harm.

In the interpretability analysis of the Physical Harm prediction model, Human Rights Violations emerged as the most dominant feature. However, its influence on Physical Harm exhibits a suppressive effect. This phenomenon may stem from the tendency of news cases involving Physical Harm to overlook Human Rights Violations, and conversely, cases centered on Human Rights Violations are less likely to include instances of Physical Harm. Based on the SHAP results, it can also be concluded that Physical Harm primarily impacts individuals. Errors during the Model Building phase are identified as the most significant contributors to the occurrence of Physical Harm among all lifecycle attributes.

For modeling Economic Loss, SHAP analysis reveals that User Experience and Interaction is the lifecycle stage with the greatest impact on economic loss. In other words, issues arising during the User Experience and Interaction phase are the most likely to lead to economic losses. Additionally, Privacy Violation is identified as another significant contributing factor. The occurrence of Privacy Violations also contributes to Economic Loss, highlighting the interconnected nature of privacy breaches and their financial consequences.

Based on the above results, we are able to provide a more in-depth interpretation of the cases presented in Table 2. Based on the SHAP-based interpretability results, this event presents a high likelihood of equal rights violations and psychological harm. The presence of severe psychological impact, involvement of vulnerable groups, and influence on self-identity align with the top SHAP features identified in both the equal rights and psychological harm prediction models. Moreover, the fact that the harm was triggered by untimely maintenance of training data further strengthens the model’s prediction, as this feature consistently shows a strong positive SHAP value in the psychological harm model. The engagement of early AI lifecycle stages—especially data acquisition and preprocessing—also contributes significantly to both harm dimensions, supporting the interpretation that this event is a compound ethical issue centered on representational bias and identity-based harm.

## Discussion

The empirical findings directly substantiate and operationalize the conceptual priorities delineated in the background. First, the prominence of discrimination-related victimization (Fig. 4e) and the positive SHAP contributions of psychological harm variables to equal rights violations corroborate the centrality of bias as a major ethical hazard. Second, the finding that over half of all incidents exhibit global transmissibility via social media channels (Fig. 11) echoes the background’s emphasis on the amplification of fake news, confirming that rapid, transboundary diffusion constitutes a systemic risk vector.

From a macro–micro integration perspective, the observed longitudinal growth of incidents after 2013 (Fig. 4a) and their uneven geospatial clustering (Fig. 4b) translate individual news reports into statistically robust temporal–spatial patterns, thus achieving the scale-bridging objective proposed in the background. Simultaneously, SHAP-based feature attribution analysis (Fig. 15) reconnects these macro patterns to micro-level characteristics, such as lifecycle stage and data maintenance quality, thereby fulfilling the study’s call for integrated multi-scale analytics.

The background assigns a pivotal governance role to AI practitioners and major technology providers; empirically, 49.86% of incidents implicate five dominant providers (Fig. 4c), and “limitations of traditional oversight” emerges as the leading root cause in 55.85% of cases (Fig. 12). These findings concretize the practitioner-centric risk hypothesis and underscore the oversight deficiencies identified earlier.

Methodologically, the background advocates the coupling of ontological structuring with explainable machine learning approaches. The high predictive performance of the XGBoost–SHAP models (Table 3), along with the interpretable ranking of lifecycle and harm-related features, validates this methodological recommendation, demonstrating how transparent modeling can effectively reveal key risk drivers (e.g., “Data Acquisition & Pre-processing” as the principal determinant of privacy violations).

Finally, lifecycle stage analysis shows that 73.79% of incidents arise during the operation and supervision phases (Fig. 13), supporting the background’s assertion that continuous post-deployment monitoring is essential, beyond design-phase interventions alone. These findings, which align with the ontology structure, were further validated through explainable machine learning, thereby supporting the effectiveness of our ontological model.

These empirical findings offer quantitative evidence supporting each thematic strand outlined in the background—bias, misinformation propagation, practitioner responsibility, macro–micro synthesis, and interpretability—thereby reinforcing the study’s theoretical contributions and addressing key knowledge gaps. For policymakers, the results underscore the importance of regulatory frameworks that mandate regular model updates and embed human rights considerations in AI governance. For industry practitioners, they highlight the need for stronger internal safeguards, particularly in data maintenance, user interaction oversight, and privacy protection. For standard-setting bodies, the analysis points to the value of developing lifecycle-oriented standards, with emphasis on risks emerging during model development and deployment. Overall, the interpretability analysis provides a robust empirical foundation for designing more transparent, responsible, and resilient AI systems.

This study still has several limitations. First, the dataset size is limited. As of the time of this research, the sample size was restricted to 500. There are two main reasons for the relatively small sample size. First, at the beginning of this study, the number of cases we were able to retrieve from publicly available news datasets was limited. Second, the recoding and validation processes required substantial human effort. While the small dataset may limit the generalizability of case coverage, it provides a solid foundation for demonstrating the feasibility of the proposed ontological model, as well as the visualization and explainability analysis methods. In addition, news data inevitably carries certain biases, even though the sources we employed have undergone preliminary validation procedures. In practice, our dataset is primarily derived from major public news platforms, which to some extent ensures both the accessibility and credibility of the information^32,33^. Nonetheless, the systematic auditing of news-based data and the mitigation of its inherent biases remain important directions for future research. With regard to the ethical considerations of the framework, we incorporated manual review during the annotation process and employed explainable AI techniques not only for interpretability but also for bias detection. Moreover, the framework is intended to function as a diagnostic and exploratory tool, designed to be used in conjunction with human judgment and policy deliberation. Responsible use of the framework requires critical reflection on both the input data and the interpretive outputs generated by the model.

The visual analytics conducted in this study were all based on the complete dataset, which inevitably limited the granularity of analysis at the level of individual cases or specific case clusters. This represents a notable limitation and an important direction for future improvement. We will also explore new techniques to improve both the quality and volume of the data. Second, despite the data validation process, manual annotation inevitably introduces errors. How to better address these errors during the data validation and preprocessing stages requires further exploration. Finally, in terms of analytical methods, further exploration of innovative approaches suitable for small sample sizes is necessary to unlock their potential.

## Conclusions

In this article, we firstly introduced a pioneering approach to modeling risks of AI events using a standardized ontological risk model. This model allows us to categorize various types of AI risks within a unified framework and facilitate a systematic visual analysis of these risks. Based on this model, we generated a recoded dataset with enriched attributes. This dataset can not only enhance a holistic comprehension of the nature of AI risks, but also enables systematic examination of how different AI risks are interrelated. Based on explainable machine learning, we identified potential driving factors behind several common attributes of AI risks. Our analytical discussion provides a foundation for decision-makers to support more informed policy development and standard setting.

## Data Availability

The datasets used and analyzed during the current study available from the corresponding author (Peng Luo; peng.luo@tum.de) on reasonable request.

## Abbreviations

AI: Artificial intelligence
NLP: Natural language processing
KPI: Key performance indicators
XGBoost: Extreme Gradient Boosting
SHAP: Shapley additive explanations
